# Supplementary material for: The Systems Biology Research Tool: evolvable open-source software
Source: BMC Syst Biol. 2008 Jun 29;2:55. doi: 10.1186/1752-0509-2-55 (PMC2446383; doi:10.1186/1752-0509-2-55)
Supplement: Additional file 1 — SBRT Archive. An archive of the current version of the Systems Biology Research Tool. [file 1752-0509-2-55-S1.zip › sbrt-1.4.0/doc/developers_guide/api/sbrt/shell/mng/algebra/package-tree.html]

sbrt.shell.mng.algebra Class Hierarchy


|  |  |  |
| --- | --- | --- |
| **Overview** \| **Package** \| Class \| Use \| **Tree** \| **Deprecated** \| **Index** \| **Help** | |  |
| **PREV**   **NEXT** | **FRAMES**    **NO FRAMES**     **All Classes** |  |


---


## Hierarchy For Package sbrt.shell.mng.algebra

**Package Hierarchies:**: All Packages

---

## Class Hierarchy

- java.lang.Object
  - sbrt.shell.mng.**AbstractProcessManager** (implements sbrt.shell.mng.ProcessManager<K,V>)
    - sbrt.shell.mng.algebra.**LinearCombConverterManager** (implements sbrt.shell.mng.ShellProcessManager<K,V>)- sbrt.shell.mng.algebra.**LinearSolverManager** (implements sbrt.shell.mng.KernelProcessManager<K,V>)- sbrt.shell.mng.algebra.**MatrixConverterManager** (implements sbrt.shell.mng.ShellProcessManager<K,V>)- sbrt.shell.mng.algebra.**MultipleVectorsConverterManager** (implements sbrt.shell.mng.ShellProcessManager<K,V>)- sbrt.shell.mng.algebra.**SingleVectorConverterManager** (implements sbrt.shell.mng.ShellProcessManager<K,V>)

---


|  |  |  |
| --- | --- | --- |
| **Overview** \| **Package** \| Class \| Use \| **Tree** \| **Deprecated** \| **Index** \| **Help** | |  |
| **PREV**   **NEXT** | **FRAMES**    **NO FRAMES**     **All Classes** |  |


---
